# A Natural Dietary Flavone Myricetin as an α-Hemolysin Inhibitor for Controlling *Staphylococcus aureus* Infection

**DOI:** 10.3389/fcimb.2020.00330

**Published:** 2020-07-23

**Authors:** Tingting Wang, Peng Zhang, Hongfa Lv, Xuming Deng, Jianfeng Wang

**Affiliations:** ^1^Department of Thoracic Surgery, The First Hospital of Jilin University, Changchun, China; ^2^Key Laboratory of Zoonosis Research, Ministry of Education, Institute of Zoonosis, College of Veterinary Medicine, Jilin University, Changchun, China

**Keywords:** myricetin, *Staphylococcus aureus*, α-hemolysin, anti-virulence, anti-infection, inflammation

## Abstract

*Staphylococcus aureus*, an important agent for lethal bacterial infections, can cause a broad spectrum of diseases in various host species. The emergence of multidrug-resistant and highly virulent strains has raised increasing concerns about the novel therapeutic strategies or agents available for treating *S. aureus* infection. The critical role of Hla, an essential virulence determinant, in the pathogenicity of *S. aureus* renders this toxin an attractive target for effective therapeutic applications. Here, we have identified myricetin as an effective inhibitor of Hla that simultaneously inhibits Hla production and neutralizes Hla activity without affecting bacterial growth. Myricetin treatment reduced the oligomerization of Hla and Hla-mediated biofilm formation. The addition of myricetin to the coinfection system of host cells and *S. aureus* significantly decreased cell injury and downregulated the inflammatory response in cells. Furthermore, *S. aureus*-infected mice that received myricetin showed alleviated tissue damage in the lung. Our results indicated that myricetin inhibits *S. aureus* virulence by targeting Hla and downregulates the inflammatory response in host cells. Overall, in addition to traditional antibiotics with antibacterial activity, myricetin may represent a potential candidate, and strategy for *S. aureus* infection.

## Introduction

*Staphylococcus aureus* (*S. aureus*) is a critical pathogen that causes a wide spectrum of infections, such as pneumonia, soft tissue infections, wounds, arthroses, and skin infections. The rapid spread of multidrug-resistant and highly virulent *S. aureus* strains has resulted in increased morbidity and mortality and great economic loss worldwide. Recurrent infections and the overuse of antibiotics contribute to the development of antibiotic resistance, which in turn promotes the spread of *S. aureus* (Sampedro et al., 2014). Meanwhile, this pathogen is capable of forming biofilms in stressful environments and of protecting active cells from the effects of antibiotics and host defense mechanisms. Thus, the frequency of *S. aureus* infection is increasing, but the available therapeutics are limited.

In addition to evolving various resistance mechanisms, *S. aureus* also expresses multiple virulence determinants, such as enterotoxins, sortase, hemolysins, and bicomponent leukocidins, for the invasion or modulation of natural host defense mechanisms and the establishment of infection. These virulence factors have been reported to contribute to the pathogenicity of *S. aureus* by acting in combination; however, some toxins alone can be sufficient for such contributions. Among these virulence factors, α-hemolysin (Hla) is a toxin with an indispensable role in various infections, such as pneumonia and skin abscesses (Kennedy et al., 2010). Hla is encoded by a single gene (*hla*) and is secreted as a water-soluble monomer that is approximately 33 kDa. When Hla attaches to target cells, such as platelets, epithelial cells, endothelial cells, or leukocytes, the monomers undergo conformation changes and oligomerize, forming β-barrel pores 1–2 nm in diameter through the lipid bilayer, which results in cell death and tissue lesion (Los et al., 2013). Recently, the metalloproteinase ADAM10 was proposed to be a unique receptor of Hla, and subsequent studies showed that ADAM10 is an indispensable mediator for Hla binding to various types of cell membranes and forming β-pores. Additionally, ADAM10 also contributes to barrier disruption by promoting the cleavage of E-cadherin during *S. aureus* infection (Inoshima et al., 2011).

Hla is involved in the activation of immune signaling through various means during *S. aureus* infection, including through Hla-ADAM10-mediated cytotoxicity. Hla coupled with other *S. aureus*-derived molecules can directly trigger inflammation by activating recognition receptors, such as TLR2. Indirectly, the ADAM10-mediated pore conformation of Hla causes ion fluxes that are responsible for many intracellular signaling pathways during *S. aureus* infection. The extracellular Na^+^ influx and K^+^ efflux of cells is sufficient to induce the involved immune signaling pathways, including the p38-MAPK, NLRP3-mediated, and c-Fos signaling pathways, stimulating the production of IL-1β, TNF-α, IL-6, and other cytokines (Seilie and Bubeck Wardenburg, 2017). Additionally, the Ca^2+^ signaling that precedes cell death is initiated by the disruption of the plasma membrane. However, the inflammation resulting from bacterial infection is a double-edged sword. The contribution of inflammation is dependent on the context and site of infection, which can be protective or detrimental to the host. Excessive inflammation may lead to tissue lesions and lethality. Previous studies have shown that inhibiting excessive inflammatory signaling is an alternative solution to promote *S. aureus* clearance (Gonzalez-Juarbe et al., 2015). In contrast, insufficient inflammation may be beneficial for bacterial growth and lead to severe infection. Thus, it is important to balance inflammatory reactions and bacterial infection.

Myricetin is a well-characterized natural flavonoid that widely exists in vegetables, fruits, and some beverages (Hertog et al., 1992; Mu et al., 2016); the major sources of myricetin are vegetables, fruits, and tea (Hertog et al., 1993). Myricetin was previously reported as a promising preventive natural compound with anti-inflammation, antitumor, antiviral, antibacterial, and antivirulence properties (Shih et al., 2009; Phillips et al., 2011; Ding et al., 2012; Tsai et al., 2015; Lopes et al., 2017; Silva et al., 2017). With the development of nutrition, some dietary bioactive components in food have become increasingly attractive, among which is tea, which naturally triggered our interest in researching the biological activities of myricetin. Here, we illustrated that myricetin is an effective inhibitor of Hla with the potential to protect A549 cells *in vitro* and alleviate lung injury *in vivo* during *S. aureus* infection. Additionally, studies with immune cells revealed that myricetin influences the Hla-mediated activation of immune signaling and inflammation. Thus, myricetin is proposed to be an effective anti-infection inhibitor against *S. aureus* by targeting Hla.

## Materials and Methods

### Bacterial Strains and Cell Lines

The strains used in this study were wild-type *Staphylococcus aureus* strain NCTC 8325-4 and the *hla*-deficient mutant DU1090, which were cultured in TSB (tryptic soy broth) broth or on TSB agar as previously described (Ragle and Juliane, 2009). Bacteria picked from a single colony were cultured overnight in 2 ml of TSB with shaking at 180 r/min and 37°C. The cultures were then diluted 1:100 in 20 ml of TSB for subculture under the same conditions until the OD600 reached 1.5 and 0.6 for cell infection and animal infection assays, respectively. The cultures were centrifuged (12,000 rpm, 1 min), and the bacterial pellet was collected for the infection assay. Human alveolar epithelial A549 cells were cultured in DMEM supplemented with 10% fetal serum at 37°C in 5% CO_2_. Primary peritoneal macrophages were extracted from male C57BL/6 mice as previously described (Zhang et al., 2017). Briefly, mice were injected intraperitoneally with 2 ml of 3% thioglycolate medium (BD Bioscience, USA) 3 days before cells were isolated, and then the euthanized mice were injected intraperitoneally with 5 ml of RPMI medium to perform peritoneal lavage. The macrophages were collected by centrifugation (1,200 rpm, 5 min) and cultured in RPMI supplemented with 10% FBS at 37°C in 5% CO_2_.

### Reagents and Antibodies

Myricetin (≥98%) purchased from Herbpurify (Chengdu, China) was dissolved in dimethyl sulfoxide (DMSO, Sigma-Aldrich) under sterile conditions to make a stocking solution of 40 mg/ml. The T-PER Tissue Protein Extraction Reagents and Pierce™ BCA Protein Assay Kit (Thermo Scientific, USA) were used for total protein extraction and the measurement of total protein concentration, respectively. The antibodies used in this study were as follows: polyclonal rabbit anti-ERK1/2 (Proteintech), phospho-ERK1/2 (Thr202/Thr204) (Arigo), JNK (Proteintech), phospho-SAPK/JNK (Thr183 (221)+Thr185 223) (Arigo), p38 MAPK (Cell Signaling Technology), phospho-p38MAPK (Thr180/Tyr182) (Cell Signaling Technology), NF-κB p65 (Cell Signaling Technology), phospho-NF-κB p65 (Ser536) (Cell Signaling Technology), IKK alpha, phospho-IKK alpha (Thr23) (Arigo), and monoclonal mouse anti β-actin (Proteintech) for inflammation reaction analysis and Staphylococcal α-toxin (Sigma-Aldrich) and HRP-conjugated secondary antibodies (Proteintech) for Hla production analysis. All of these antibodies were used as recommended by the manufacturers.

### Purification of Recombinant Hla

Wild-type Hla was expressed from a pET28a-based expression plasmid in *E. coli* BL21 (DE3) as previously described (Qiu et al., 2016). Briefly, *E. coli* was grown in LB medium until the OD600 reached 0.6–0.8 and induced by IPTG (isopropyl thio-D-galactopyranoside) at a final concentration of 0.3 mM at 16°C for 18 h. Bacterial cells were harvested by centrifugation at 4,000 rpm for 30 min and lysed by sonication in the presence of PMSF (phenylmethylsulfonyl fluoride). Then, the mixture was centrifuged at 12,000 rpm for 1 h at 4°C to obtain the soluble fraction. His-tagged Hla was purified with Ni^2+^-NTA beads (Qiagen) and eluted with PBS containing 200 mM imidazole. Purified protein was then dialyzed in dialysis buffer (25 mM Tris-HCl, 150 mM NaCl, 10% glycerol, 1 mM DTT, pH 7.5) to remove imidazole and was stored at −80°C.

### Hemolysis Assay

A rabbit erythrocyte hemolysis assay was applied to screen for effective inhibitors of Hla. Briefly, 1 μl of recombinant Hla was added to 975 μl of PBS containing various concentrations of the designated compounds, and then the mixtures were preincubated at 37°C for 15 min. A total of 25 μl of freshly washed rabbit erythrocytes was added to each tube, and the reaction mixtures were incubated for another 15 min at 37°C. Finally, the reaction mixtures were centrifuged (12,000 rpm, 1 min) to remove the cell debris. The hemoglobin released in the cell-free supernatants was quantified spectrophotometrically at OD543 to assess the effect of those compounds on the hemolytic activity of Hla. The sample treated with DMSO was regarded as a negative control, and the sample treated with Hla and DMSO served as a positive control (100% hemolysis). The hemolysis was defined as the ratio of the OD543 value of each sample relative to the positive control.

### Susceptibility Assays

The minimal inhibitory concentration (MIC) of myricetin on *S. aureus* was determined by the broth microdilution method as previously described (Irith et al., 2008). The minimum concentration of myricetin that inhibited *S. aureus* growth was regarded as the MIC. To assess the effect of myricetin on the growth of *S. aureus*, a growth curve was performed. Briefly, *S. aureus* was grown in TSB supplemented with the indicated concentrations of myricetin and 37°C. The growth curve was monitored by measuring the OD600 at an interval of 30 min until the culture reached the stationary phase. To verify whether myricetin inhibited Hla expression, the supernatants and precipitates of the bacterial culture were collected at the last interval, and a hemolysis assay, as described above, and a western blot assay were performed. The sample without myricetin was treated with DMSO.

### Western Blot Assay

Supernatants and precipitates were denatured with 5 × protein loading buffer and 1 × protein loading buffer, respectively, at 100°C for 8 min. Following separation by 10% SDS-PAGE, the proteins of interest were transferred onto polyvinylidene membranes. After blocking with 5% (w/v) skimmed milk in TBST for 2 h at room temperature, the membranes were incubated with the rabbit *Staphylococcal* α-toxin antibody (diluted 1/1000 in TBST) overnight at 4°C, washed in TBST 3 times for 10 min each, and incubated with HRP-conjugated goat anti-rabbit antibody (diluted 1/5000 in TBST) at room temperature for 1.5 h, followed by the same wash step. Finally, the signals were obtained with an ECL Plus Western Blotting Detection System (Tanon). The gray values of the western blotting bands were procured using Image-Pro Plus 6.0 software.

### Oligomerization Assay and Circular Dichroism Analysis

An oligomerization assay was performed as previously described to illustrate whether myricetin could interfere with the formation of pores on the cell membrane (Wang et al., 2016). Briefly, 2.5 μg of purified his-tagged recombinant Hla was incubated with 5 mM deoxycholate in PBS buffer with or without myricetin at 22°C for 25 min. Then the reaction mixtures were treated with 5 × protein loading buffer without β-mercaptoethanol at 55°C for 10 min. The Hla oligomers and monomers were detected by western blot assay as described above. To assess whether myricetin changes the secondary structure of Hla, circular dichroism assay was performed. Briefly, purified Hla was diluted to 0.5 mg/ml in PBS in the presence of myricetin or DMSO; after an incubation of 37°C for 15 min, samples were then subjected to circular dichroism assay with a CD spectrophotometer (MOS-500; Bio-Logic, France).

### *In vitro* Biofilm Formation Assay

To investigate the effect of myricetin on Hla-dependent biofilm formation, an *in vitro* biofilm formation assay was performed according to a previous report with slight modification (Anderson et al., 2018). In brief, an overnight culture of *S. aureus* was diluted 1:100 in 96-well-microtiter plates containing TSB supplemented with the indicated concentrations of myricetin and incubated at 37°C for 36 h. Following a gentle wash to remove the unattached cells, the adherent cells were dried at 60°C for 10 min and then stained with 0.1% crystal violet at room temperature for 1 h. Then, the cells were gently washed a few times until the water became clear, and the fixed crystal violet in the wells was solubilized in ethanol with shaking at room temperature for 10 min. The biofilms were quantified by measuring the OD600 of each well. The sample without myricetin was treated with DMSO.

### Infection of A549 Cells

A549 cells were seeded in 96-well-plates at a density of 1.5 × 10^4^ cells per well the day before infection. The *S. aureus* suspension described above was added to the cells at a multiplicity of infection (MOI) of 50 in the presence of myricetin at the indicated concentrations, and the 96-well-plates were incubated at 37°C for 6, 16, or 24 h. The LDH released into the supernatants was measured with a Cytotoxicity Detection Kit (LDH, Roche) as recommended by the manufacturer. Cells at the bottom of the wells were stained with live/dead reagent (Invitrogen) and observed under an inverted fluorescence microscope (Olympus).

### Infection of Primary Peritoneal Macrophages

Primary peritoneal macrophages from C57BL/6 mice were seeded in 6-well-plates at a density of 4 × 10^6^ cells per well the day before infection. The *S. aureus* suspension described above was added to the cells at a multiplicity of infection (MOI) of 5 in the presence of myricetin at the indicated concentration, and the 6-well-plates were incubated at 37°C for 5 h. Following centrifugation, the cytokines (IL-1β, IL-6, and TNF-α) in the supernatants were detected using mouse ELISA kits (BioLegend) as recommended by the manufacturer. Three biological replicates were carried out. In addition, the total proteins were extracted using T-PER Mammalian Protein Extraction Reagent and the inflammatory mediators were evaluated by western blot assay as described above. Antibodies P65, P-P65, IKK-α, P- IKK-α, P38, P-P38, ERK, P-ERK, JNK, P-JNK, NLRP3, and β-actin were diluted 1/1000 in TBST. HRP-conjugated goat anti-rabbit antibody and HRP-conjugated goat anti-mouse antibody were diluted 1/5000 in TBST.

### Murine *S. aureus* Infection

C57BL/6 female mice aged 6–8 weeks (20–25 g weight) were purchased from the Jilin University Experimental Animal Center and received humane care in compliance with the guide by the Jilin University Institutional Animal Care Committee. All animal assays were approved by this committee.

For *S.aureus* infection, 50 ml of culture aliquot of the NCTC 8325-4 strain (OD600 = 0.6) was centrifuged and the bacterial pellet was washed in PBS 3 times, then the pellets were resuspended in 1 ml PBS. Twenty microliter of this suspension containing 3 × 10^8^ CFUs was nasally delivered to the mice for infection. Mice were grouped as follows: positive group (*n* = 5), infected mice left untreated; myricetin group (*n* = 5), myricetin administered in vehicle to infected mice (100 mg/kg) at 8 h intervals; vehicle group (*n* = 5), only vehicle administered to uninfected mice. At 48 h post-infection, mice were anesthetized, and the left lungs were fixed in 10% formalin to perform histopathological analysis via hematoxylin and eosin (H&E) staining analysis (*n* = 3). H&E stained slides were examined by a professional pathologist using a microscope (Olympus, Japan) and histopathology scores were determined as described previously (Pan et al., 2016).

### Statistical Analysis

All data are presented as the mean ± SD (*n* ≥ 3). The statistical analyses were performed by One-way ANOVA followed by the Newman-Keuls test. ^*^p < 0.05 and ^**^p < 0.01.

## Results

### Myricetin Simultaneously Inhibits Hla Hemolytic Activity and Production Without Affecting *S. aureus* Viability

**Figure 1:**
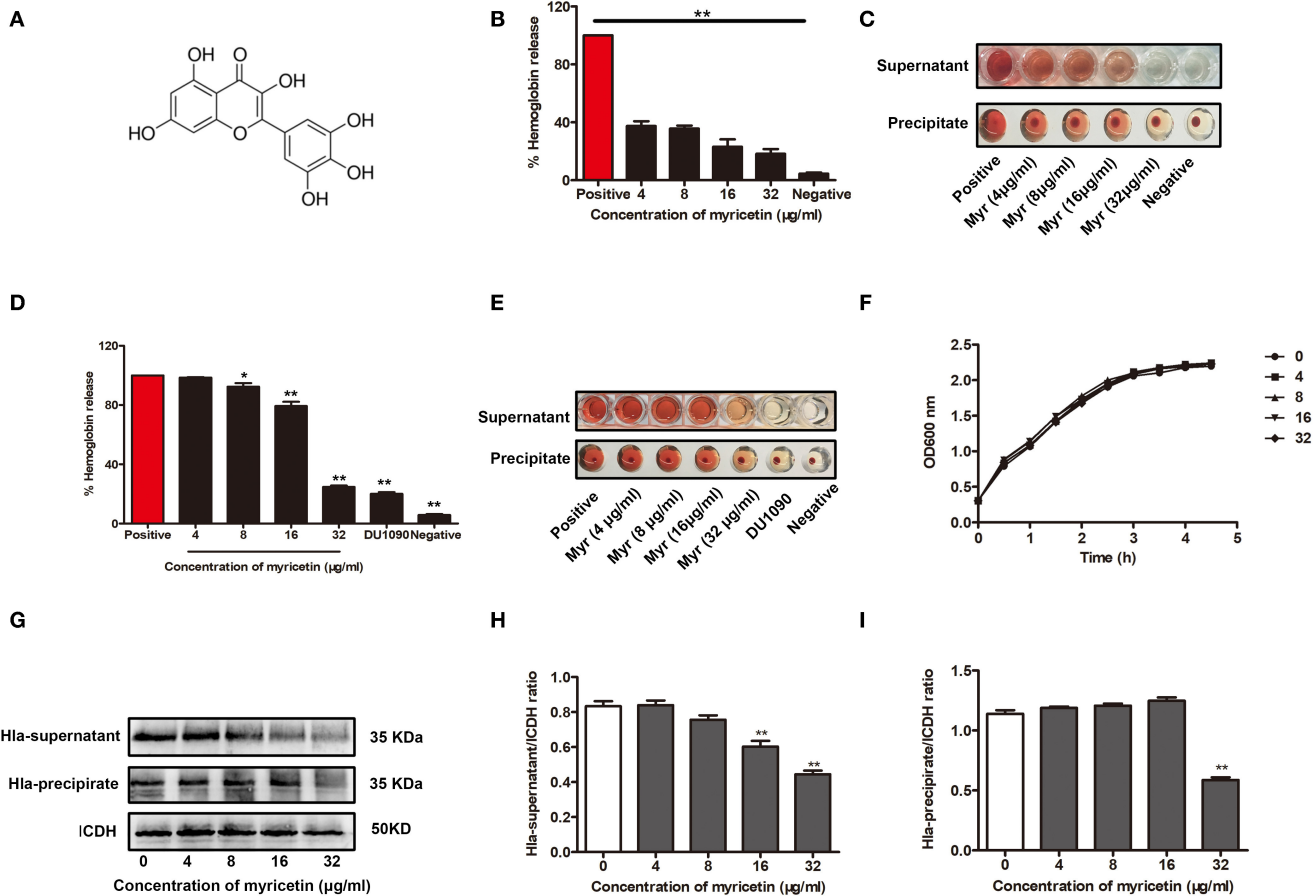
Myricetin inhibits the hemolytic activity of Hla. **(A)** Chemical structure of myricetin. **(B,C)** Inhibition of Hla activity by myricetin (Myr). The hemolytic activity of purified Hla (5 μg/ml) pretreated with the indicated concentrations of myricetin was determined using a hemolysis assay. **(D,E)** Hemolytic activity of the supernatants from *S. aureus* co-cultured with myricetin. The sample treated with DMSO was regarded as a negative control, and the sample treated with Hla or the supernatants from 8325-4 strain in the presence of DMSO served as a positive control (100% hemolysis). **(F)** Growth curves of *S. aureus* in the presence of various concentrations of myricetin. **(G)** Western blot assay for Hla expression in the supernatants and precipitates of *S. aureus* cultures treated with or without myricetin. The gray value of Hla expression in the culture supernatants **(H)** and precipitate **(I)** of *S. aureus* cultures treated with or without myricetin using Image-Pro Plus 6.0 software. Each column shows the mean ± SD of three independent experiments and analyzed by the one-way ANOVA. *P < 0.05 and **P < 0.01 compared to the positive group.

Myricetin (Figure 1A) has been shown to have multiple biological activities, including antioxidant, anti-inflammatory, antimicrobial, and cytoprotective activities (Hu et al., 2018; Kim et al., 2018; Rocha et al., 2018). Here, the addition of myricetin significantly reduced the hemolytic activity of purified Hla. Statistically, the lysis of rabbit erythrocytes caused by Hla was attenuated by this compound in a dose-dependent manner, and the hemolytic activity of Hla was reduced from 100% to 37.4% ± 1.041 in the presence of 4 μg/ml myricetin. Furthermore, the hemolytic activity of Hla was almost completely abrogated when treated with 32 μg/ml myricetin (Figures 1B,C). As expected, such inhibition was also observed for the hemolytic activity of culture supernatants of *S. aureus* co-cultured with various concentrations of myricetin (Figures 1D,E), suggesting that myricetin treatment may directly neutralize Hla activity, inhibit *S. aureus* growth, or reduce Hla production. Consistent with our hypothesis, treatment with myricetin at the concentrations required for the inhibition of Hla activity did not visibly inhibit *S. aureus* viability (Figure 1F). However, myricetin treatment remarkably reduced the production of Hla at the concentrations that didn't affect *S. aureus* growth (Figures 1G–I). Additionally, the MICs of myricetin tested for *S. aureus* were >256 μg/ml, which greatly exceeded the test concentration in all of the assays. Taken together, these data revealed that myricetin effectively inhibits Hla by simultaneously reducing Hla hemolytic activity and production without affecting *S. aureus* growth.

### Myricetin Inhibits the Deoxycholate-Induced Oligomerization of Hla by Changing the Secondary Structure of Hla

**Figure 2:**
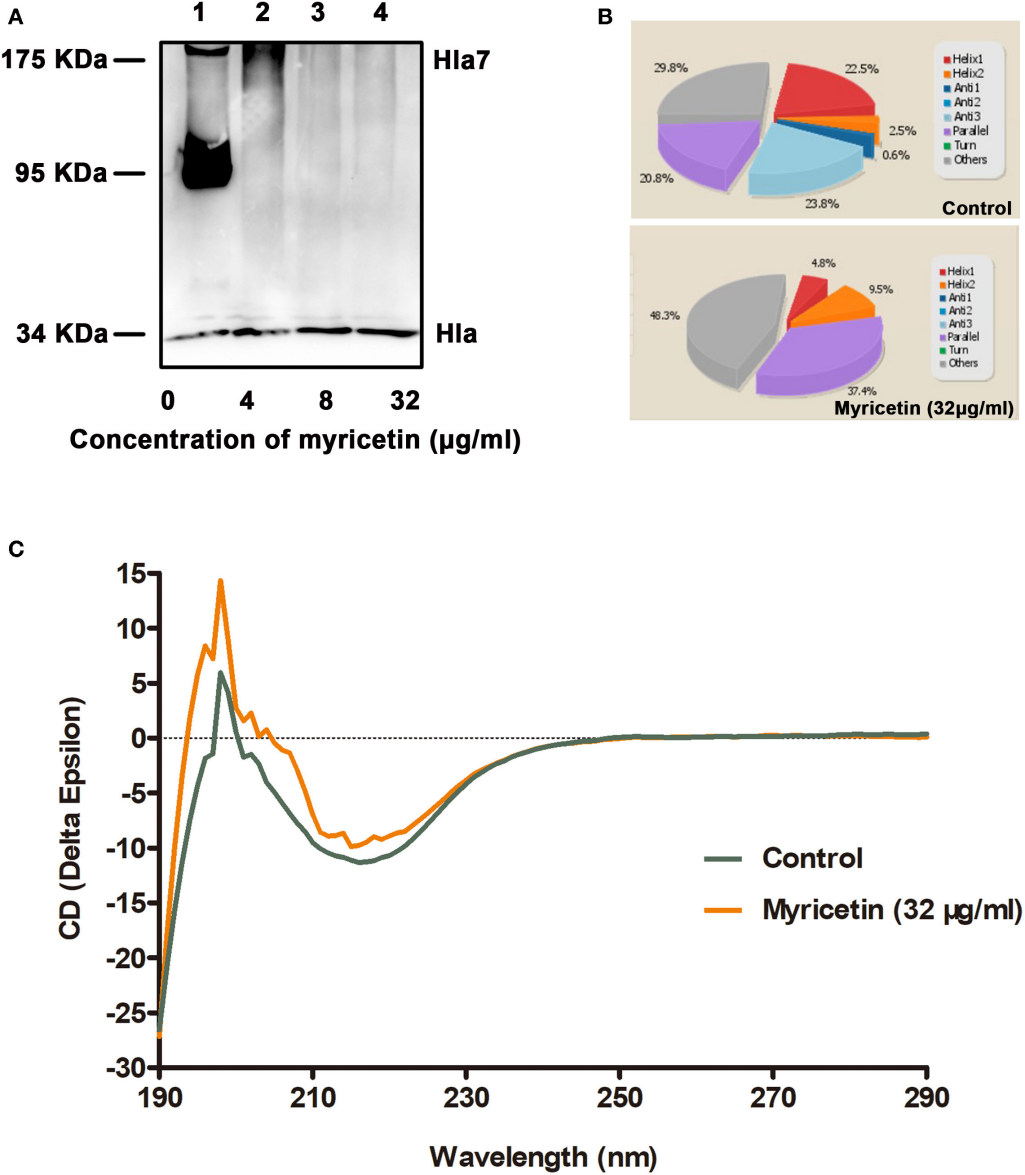
Myricetin interferes with the deoxycholate-induced oligomerization of Hla. **(A)** The oligomerization of Hla in the presence of various concentrations of myricetin was determined using western blot assay. **(B,C)** Circular dichroism analysis of Hla in the presence or absence of myricetin. The percentages of α-helix and turn conformations in Hla were both reduced by myricetin. The sample treated with DMSO was used as a control.

The oligomerization (heptamer formation) of Hla is critical for the pore-forming activity of the protein. Thus, an oligomerization assay was employed to further evaluate whether myricetin treatment could interfere with the formation of Hla oligomers. As expected, Hla heptamers were observed following the addition of deoxycholate without myricetin. However, heptamer formation was significantly decreased in the samples treated with various concentrations of myricetin (Figure 2A). Furthermore, obvious changes in the secondary structure of Hla were observed during treatment with myricetin based on circular dichroism, with the percentages of α-helix and turn conformations in Hla both reduced when treated with myricetin (Figures 2B,C), suggesting that a direct engagement of myricetin with Hla occurred. Thus, the interaction between Hla and myricetin reduced the heptamer formation of this toxin and subsequently inhibited the pore-formation activity of Hla.

### Myricetin Reduces Hla-Mediated Biofilm Formation in *S. aureus*

**Figure 3:**
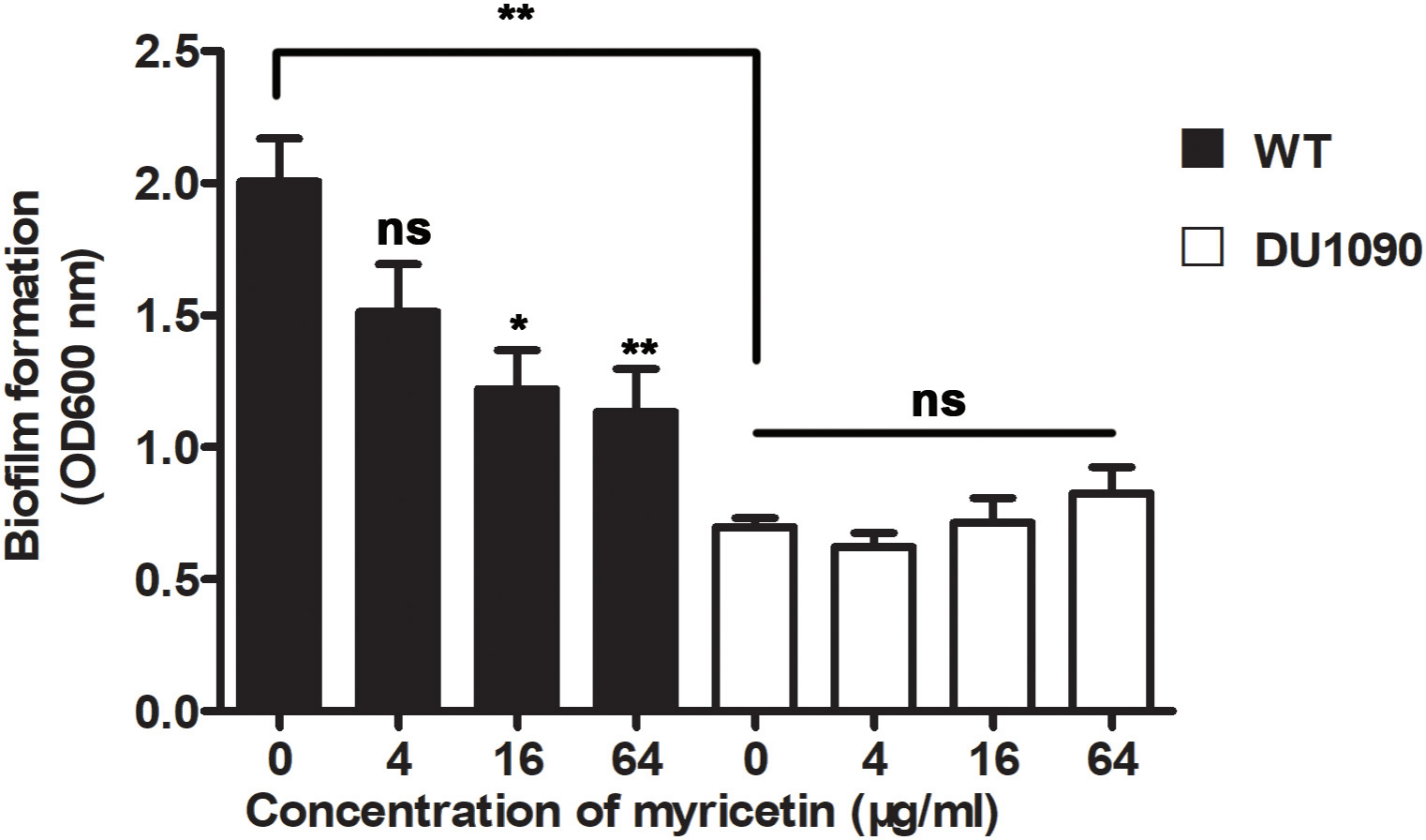
Myricetin reduces the Hla-mediated biofilm formation of *S. aureus*. The biofilms of *S. aureus* treated with myricetin were stained with crystal violet and quantified by measuring the OD600. Bars show the amounts of biofilm formation with mean ± SD (*n* = 3) and analyzed by one-way ANOVA. *P < 0.05 and **P < 0.01 compared to the positive group.

A previous study has reported that mutations in Hla lead to decreased biofilm formation in *S. aureus* (Anderson et al., 2018). In line with their finding, the WT *S. aureus* strain formed considerable biofilms in the wells (OD > 1.8), while the Hla-deletion *S. aureus* strain was defective in biofilm formation (OD < 0.8) (Figure 3). Consistent with the inhibition of Hla activity and production by myricetin, myricetin treatment significantly repressed WT *S. aureus* biofilm formation in a dose-dependent manner. Some small molecules impact biofilm formation in simple plastic *in-vitro* tests simply because they interfere with the attachment of the bacteria to the plastic surface, or diminish interbacterial interactions. However, myricetin treatment didn't affect the biofilm formation of Hla-deletion *S. aureus* strain, indicating that myricetin reduces biofilm formation in a Hla dependent way and this inhibitory effect was not attributed to the physico-chemical properties of myricetin. Thus, our findings suggested that myricetin reduces *S. aureus* biofilm formation by targeting Hla.

### Myricetin Prevents *S. aureus*-Mediated Cell Injury

**Figure 4:**
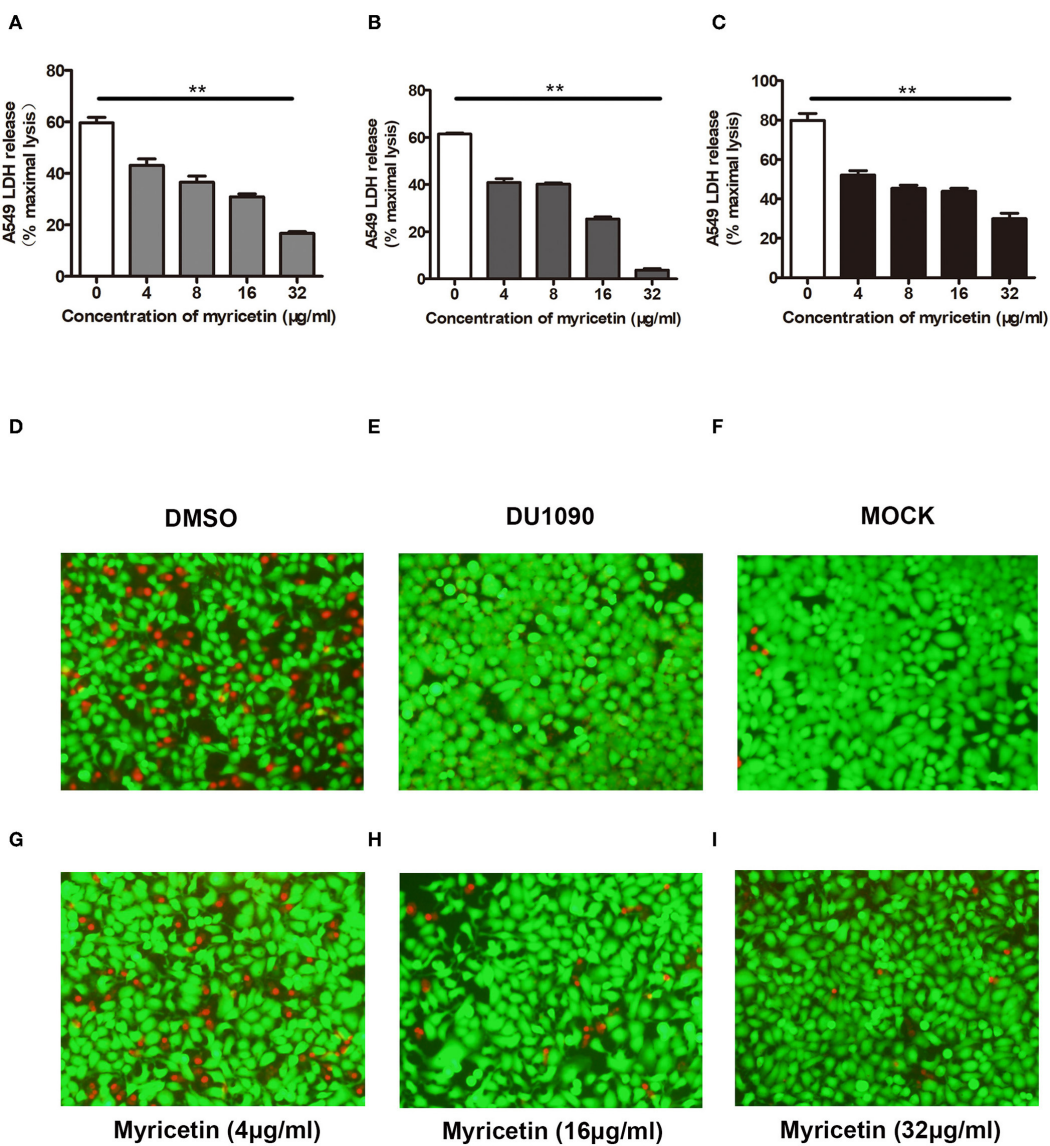
Myricetin prevents Hla-mediated cell injury during *S. aureus* infection. A549 cells infected with *S. aureus* for 6 h were stained with live (green)/dead (red) reagent, and lactate dehydrogenase released from cells was evaluated by LDH release assay. Cell cytotoxicity induced by *S. aureus* 8325-4 in the presence of the indicated concentrations of myricetin at 6 h **(A)**, 16 h **(B)**, and 24 h **(C)** post-infection was measured. The sample without myricetin was treated with DMSO. Cells infected with WT *S. aureus* **(D)** or Hla-deficient strain DU1090 **(E)** in the absence of myricetin. **(F)** Untreated cells. Cells infected with WT *S. aureus* in the presence of myricetin at the concentration of 4 μg/ml **(G)**, 16 μg/ml **(H)**, and 32 μg/ml **(I)**. Bars show the amounts of LDH release with mean ± SD (*n* = 3) and analyzed by one-way ANOVA. **P < 0.01 compared to the sample without myricetin.

A549 cells have been widely used as a valuable model to study Hla-mediated cell injury during *S. aureus* infection (Wilke and Bubeck Wardenburg, 2010). Here, A549 cells were infected with *S. aureus* to evaluate whether myricetin is capable of protecting cells. As expected, myricetin treatment remarkably reduced the release of LDH from cells infected with the WT strain 8325-4 compared to untreated cells (Figure 4A). Interestingly, such protection was observed even at 16 and 24 h post-infection (Figures 4B,C), indicating that myricetin may provide long-term protection for A549 cells against *S. aureus*. In agreement with these results, evident cell death (red fluorescence) was detected for the 8325-4-infected cells (Figure 4D), but not for the DU 1090-infected cells (Figure 4E) or the untreated cells (Figure 4F). The sample treated with increasing concentrations of myricetin showed far fewer dead cells in a dose-dependent manner (Figures 4G–I). Taken together, our results established that myricetin prevents *S. aureus*-mediated cell injury by inhibiting Hla activity and production.

### Myricetin Suppressed the Activation of the MAPK Pathway

**Figure 5:**
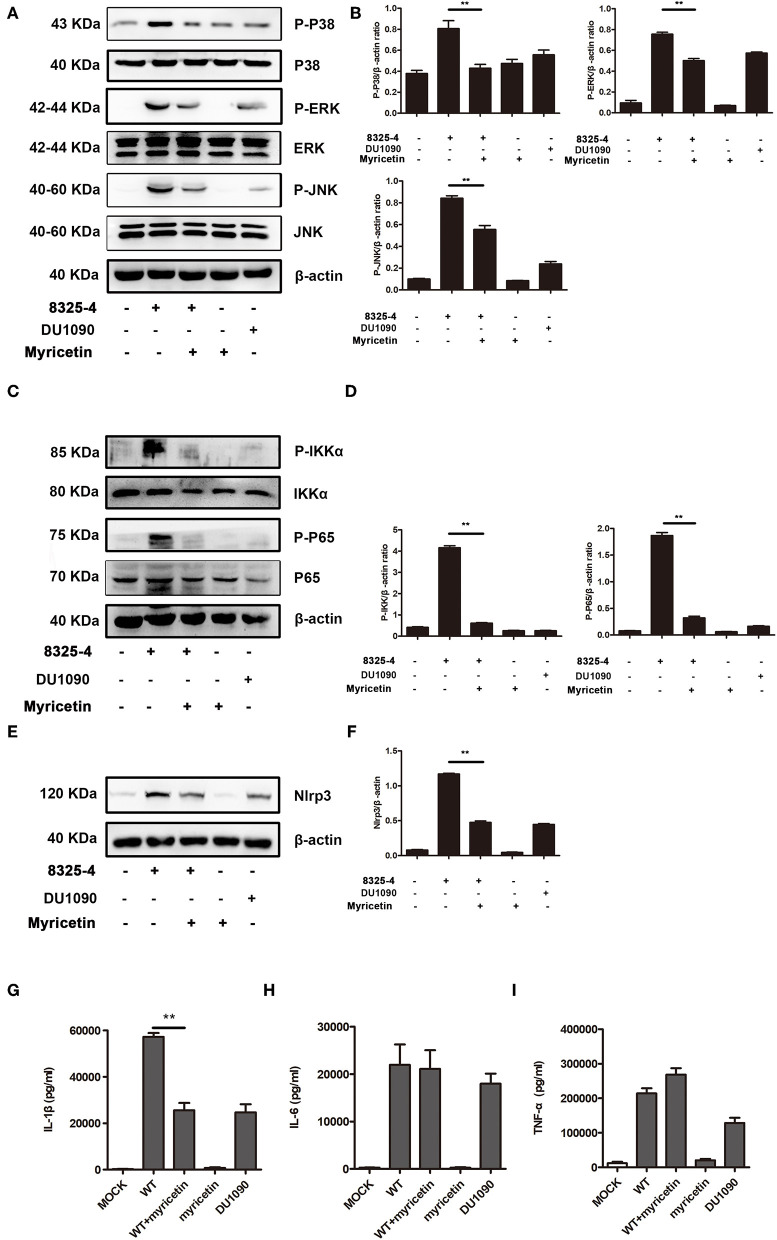
Myricetin suppressed the activation of the MAPK and NF-κB pathways stimulated by *S. aureus*. Primary peritoneal macrophages extracted from male C57BL/6 mice were infected with *S. aureus* in the presence or absence of myricetin (MOI = 5) for 5 h. The protein samples from infected cells were analyzed by western blot, and the supernatants of the coinfection system were used to perform an ELISA assay. Inhibition of the MAPK pathway **(A)**, NF-κB pathway **(C)**, and Nlrp3 inflammasome **(E)** by myricetin. The gray value of p-p38, p-ERK, and P-JNK **(B)**, p-IKK and P-P65 **(D)**, and Nlrp3 **(F)** was procured using Image-Pro Plus 6.0 software. The production of IL-1β, IL-6, and TNF-α in the supernatants of the coinfection system **(G-I)**. Bars show the levels of cytokines with mean ± SD (*n* = 3) and analyzed by one-way ANOVA. **P < 0.01 compared to the positive group.

The MAPK pathway plays an important role in inducing proinflammatory gene expression during *S. aureus* infection. Consistent with a previous study (Pauline and Pier, 2012), the MAPK signaling pathway was activated in *S. aureus* 8325-4-infected macrophages but not in the macrophages infected with the Hla-deficient strain DU 1090. Here, our data demonstrated that myricetin decreases the phosphorylation of p38, ERK, and JNK induced by *S. aureus* (Figures 5A,B). In the cells without *S. aureus* infection, myricetin treatment showed no influence on the activation of the MAPK pathway. Together, the collective results indicated that the inhibitory effect of myricetin on the MAPK pathway in *S. aureus*-infected macrophages may be attributed to the attenuation of pore formation and inhibition of Hla expression by myricetin.

### Myricetin Reduced the Activation of the NF-κB Pathway

NF-κB, an indispensable transcription factor that ubiquitously exists in multiple cell types, participates in controlling the acute immune response, and other immune processes (Zhu et al., 2014). *S. aureus* infection obviously elicited the phosphorylation level of p65 and IKK-α in mice-derived primary peritoneal macrophages; however, such phosphorylation was not visible in cells treated with the Hla-deficient strain or myricetin alone and was similar to that of the cells without any treatment. Interestingly, the addition of myricetin inhibited the phosphorylation of p65 and IKK-α in *S. aureus*-infected macrophages (Figures 5C,D). Thus, our results indicated that myricetin suppresses the Hla-mediated activation of NF-κB during *S. aureus* infection.

### Myricetin Downregulated the Release of Cytokines in Macrophages Stimulated by *S. aureus*

Hla-mediated K^+^ efflux and the activation of the NLRP3 inflammasome are responsible for inducing IL-1β secretion, and the activation of the NF-κB pathway is associated with the accelerated secretion of TNF-α, IL-6, and IL-1β (Seilie and Bubeck Wardenburg, 2017). These cytokines participate in the regulation of the host immune response during *S. aureus* infection. The increased NLRP3 inflammasome in cells induced by *S. aureus* was significantly inhibited by myricetin (Figures 5E,F). Consistent with previous studies, mice-derived primary peritoneal macrophages released a large amount of proinflammatory cytokines upon infection with WT *S. aureus*, including IL-1β, IL-6, and TNF-α (Figures 5G–I), illustrating a rapid inflammatory response, while the macrophages infected with the Hla-deficient strain or treated with myricetin alone did not have this response. Although no influence on the production of TNF-α or IL-6 was observed, myricetin treatment significantly decreased the secretion of IL-1β. Taken together, our results suggested that myricetin treatment regulates the production of cytokines by targeting Hla, which may provide protection against *S. aureus* infection.

### Myricetin Alleviates Lung Injury Caused by *S. aureus* Infection

**Figure 6:**
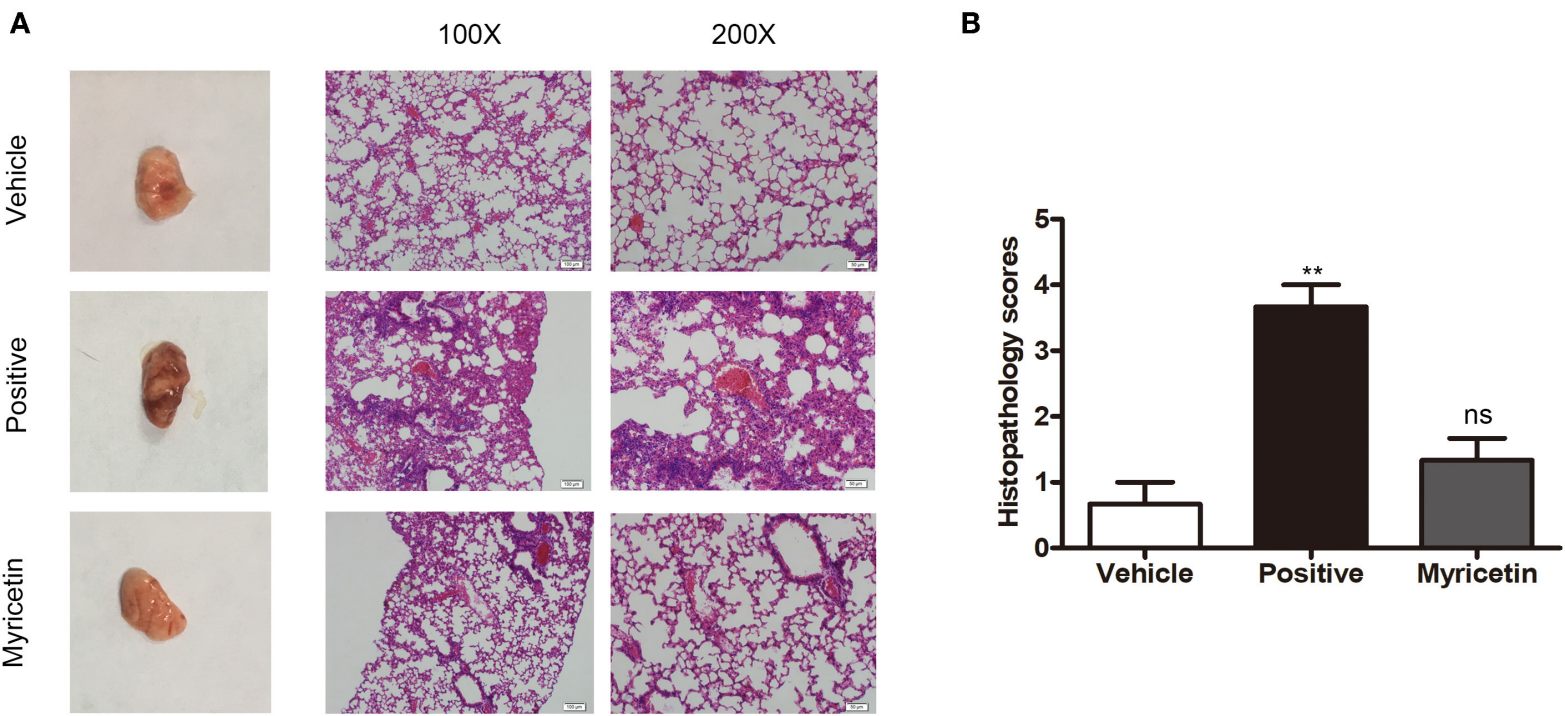
Myricetin alleviates the lung injury caused by *S. aureus*. Mice were nasally infected with 3 × 10^8^ CFU *S. aureus* 8325-4 for infection. “Vehicle” is vehicle only administered to uninfected mice (*n* = 5). “Positive” is infected mice left untreated (*n* = 5). “Myricetin” is myricetin administered in vehicle (100 mg/kg) to infected mice (*n* = 5). **(A)** The mice were sacrificed at 48 h post-infection, and the pathological injury of the lungs was analyzed by the naked eye and microscopy (*n* = 3). **(B)** Histopathology scores for lungs of mice. The data represent the mean histopathology scores for the three groups described above. Bars show the histopathology scores with mean ± SD (*n* = 3) and analyzed by one-way ANOVA. **P < 0.01 compared to the vehicle group.

The anti-*S. aureus* virulence effects of myricetin detected *in vitro* prompted us to investigate whether such protection occurs *in vivo*. Consistent with previous studies, *S. aureus* infection caused visible pathological changes of lung (Figure 6A) and higher lung injury scores than myricetin treatment or vehicle control (Figure 6B). In the positive group, the lungs were edematous with visible swelling and congestion observed by the naked eye, and the widening of the alveolar septa by edema was observed under a microscope, accompanied by variable numbers of inflammatory leukocytes and endothelial cell swelling. Treatment with myricetin remarkably alleviated pathological changes, with less inflammatory cell infiltration, swelling, and hyperemia compared to the positive group (infected, but untreated). The effect of myricetin treatment in infected mice was similar to that of the uninfected vehicle only control group. Any possible effect of myricetin on bacterial burden was not examined. Taken together, our results indicated that myricetin treatment provides visible protection against *S. aureus* infection in mice.

## Discussion

Antibiotics were the preferred choice for treating *S. aureus* infection in the early stages of antibiotic development due to their excellent antibacterial activities. However, with the rapid increase in antibiotic-resistant and highly virulent strains, such as methicillin-resistant *S. aureus* (MRSA), the disease burden is gradually increasing, and the potency of traditional antibiotics is declining. Thus, novel therapeutic strategies are urgently needed. To end this, alternative strategies have been well-explored, including antivirulence therapy, antibodies, bacteriophages, and vaccines.

Antivirulence therapy is aimed at disarming key virulence factors involved in disease progression rather than killing bacteria, meaning this strategy exerts a milder evolutionary pressure on the development of antibiotic resistance. There are many antivirulence targets for *S. aureus* due to the high adaptability, versatility, and pathogenicity of this bacteria in hosts, and it is highly attributed to the diversified virulence factors, which are employed to evade the immune system and establish infection (Diep et al., 2016). Although the combined action of multiple toxins is necessary to enhance virulence, some individual toxins may be sufficient to cause damage and inflammation. Among these toxins, Hla is indispensable for *S. aureus* infection, rendering Hla an ideal target for the development of antivirulence strategies and agents.

Here, we initially showed that myricetin attenuated the pore formation of Hla by changing the secondary structure of Hla. The hemolytic activity of Hla was reduced from 100 to 37.40% in the sample treated with 4 μg/ml myricetin. Notably, almost no hemolytic activity was observed in the sample treated with 32 μg/ml of myricetin. Interestingly, myricetin also suppressed the expression of Hla at a relatively lower concentration without affecting *S. aureus* viability. Although the mechanism for such action has not been completely characterized in our work, 32 μg/ml myricetin treatment led to 46.67% of Hla production in the supernatants and 48.46% in the precipitates of the co-cultures compared to the positive control without myricetin (Figures 1, 2). Additionally, we confirmed that myricetin inhibited the biofilm formation in a Hla-dependent way, as evident by the fact that no inhibition was observed by myricetin for the DU1090 strain (Figure 3). To evaluate whether myricetin is capable of protecting cells, we applied A549 cells to perform an LDH assay; the results demonstrated that myricetin prevented *S. aureus*-mediated cell injury by targeting Hla at a low concentration of 4 μg/ml (Figure 4).

**Figure 7:**
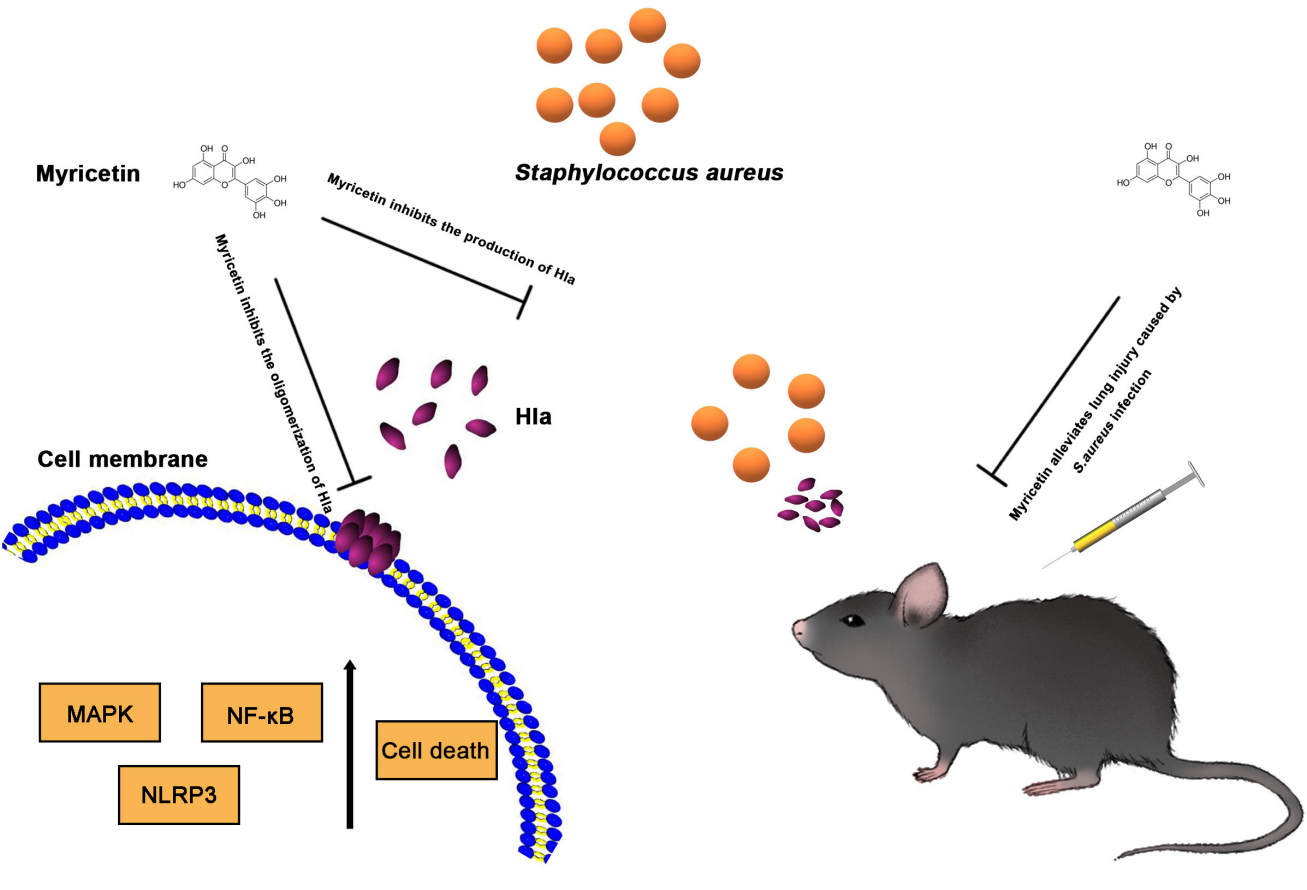
Scheme summarizing the protective effects of Myricetin on *Staphylococcus aureus* infection. Myricetin is an effective inhibitor of Hla as it simultaneously targets Hla production and Hla activity, and thereby downregulates the inflammatory response in *S. aureus* infected cells by suppressing NF-κB and MAPK signaling pathways *in vitro*, which helps alleviate lung injury *in vivo* during *S. aureus* infection.

Host innate immune responses to *S. aureus* and bacteria-associated virulence determinants are aimed at clearing bacteria and minimizing tissue damage during bacterial infection. However, excessive inflammatory responses could aggravate bacterial infection by facilitating bacterial escape from the immune system or contributing to tissue damage. For example, autophagy is regarded as a conserved pathway that confers resistance to bacteria or other pathogens (Maurer et al., 2015a,b). However, recent studies have demonstrated that autophagy is exploited by *S. aureus* to promote replication, escape, and host cell killing (Schnaith et al., 2007; Zhu et al., 2018). Hla- and IL-1β-mediated pathological injury is thought to be responsible for the high morbidity and mortality of *S. aureus* infection, especially pneumonia (Goodman et al., 2003). Here, we showed that myricetin, as an agent that targets Hla, could downregulate the cascade of host responses triggered by *S. aureus* in a Hla-dependent way and reduce the secretion of IL-1β (Figure 5), which is associated with the recruitment of immune cells, the predisposition of acute lung injury, and systemic inflammation (Goodman et al., 2003). Here, we propose that myricetin not only protects epithelial cells by inhibiting Hla activity but also reduces the enhanced secretion of IL-1β from immune cells by modulating MAPK and NF-κB pathways, and thereby alleviates lung injury *in vivo* during *S. aureus* infection (Figures 6, 7).

As a natural dietary flavonoid, myricetin has many sources, including tea, vegetables, fruits, and berries, thus, the cost of myricetin is much lower than the cost of developing new antibiotics or vaccines; myricetin has also been reported to have multiple biological activities, such as anti-inflammatory, antioxidant, ion homeostasis regulation, anti-tumor, and obesity prevention activities (Mu et al., 2016). Here, we found that myricetin protected A549 cells against *S. aureus* by targeting Hla and excessive inflammation caused by *S. aureus*, which is beneficial to relieving lung injury, and myricetin treatment did not exhibit anti-*S. aureus* activity, which will slow the development of antibiotic resistance to some extent. Although pharmacokinetic-pharmacodynamic studies were not conducted herein, our results indicate that myricetin is an attractive candidate for further testing as an adjunctive therapy for *S. aureus* infections.

With the development of antibiotics, many resistance determinants and mechanisms have gradually emerged, such as β-lactamases, enzymatic modification and inactivation, and efflux pump systems (Allen, 2008; Fast and Sutton, 2013; Vestergaard et al., 2019). Unfortunately, antibiotic resistance spreads faster than the discovery of new compounds, leading to a public health concern. The combination of some inhibitors that target β-lactamases and antibiotics is an optional first-line method for the treatment of bacterial infections (Drawz and Bonomo, 2010; Ejim et al., 2011; Wang et al., 2018). Here, we proposed that the combination of myricetin with antibiotics may improve the treatment of *S. aureus* infection and slow the development of antibiotic resistance by targeting bacterial virulence and the bacterium itself. In addition, this combination would decrease the usage of antibiotics and expand the life of antibiotics. In conclusion, myricetin is a promising candidate in the pharmaceutical industries for treating *S. aureus* infection by targeting Hla.

## Data Availability Statement

All datasets generated for this study are included in the article.

## Ethics Statement

The animal study was reviewed and approved by Jilin University Institutional Animal Care Committee.

## Author Contributions

All authors listed have made a substantial, direct and intellectual contribution to the work, and approved it for publication.

## Conflict of Interest

The authors declare that the research was conducted in the absence of any commercial or financial relationships that could be construed as a potential conflict of interest.
